# Unusual Presentation of a Congenital Ranula Cyst in a Newborn

**DOI:** 10.7759/cureus.38749

**Published:** 2023-05-09

**Authors:** A Rupesh Rao, Hemant Parakh, P Madan Mohan Rao, K Yeswanth Kumar, Ehteshaam Qadeer

**Affiliations:** 1 Pediatrics and Child Health, Datta Meghe Institute of Medical Sciences (DMIMS), Wardha, IND; 2 Neonatology, Hope Children's Hospital, Hyderabad, IND; 3 Pediatrics and Child Health, Hope Children's Hospital, Hyderabad, IND

**Keywords:** oral health management, congenital lesions, neonatal respiratory distress, plunging ranula, newborn distress

## Abstract

Congenital ranula cysts are rare, benign cysts that arise from the obstruction or rupture of the sublingual gland ducts in the oral cavity of newborns. Here, we present a case of a congenital ranula cyst in a newborn, highlighting the clinical presentation, diagnosis, and management of this rare condition. The neonate presented with a smooth, painless, and non-tender mass in the floor of the mouth, which was identified as a sublingual cyst via ultrasonography. The neonate underwent successful surgical excision of the cyst, with no complications or recurrence observed during the follow-up period. Congenital ranula cysts are a rare but treatable condition that can present in the oral cavity of newborns, and early diagnosis and surgical excision are crucial to prevent complications and ensure optimal outcomes. Healthcare providers should consider congenital ranula cysts as a differential diagnosis for any newborn presenting with a mass in the oral cavity.

## Introduction

A ranula is a cystic swelling that occurs on the floor of the mouth due to the obstruction of the sublingual gland duct. While ranulas are typically acquired, there are rare instances where they can be present at birth and are known as congenital ranula cysts. The prevalence of congenital ranula is 0.74% [1]. The term "ranula" refers to the swelling in the floor of the mouth that resembles a frog's belly. Congenital ranula cysts are typically asymptomatic and may go unnoticed until they become large enough to cause feeding difficulties, respiratory distress, or other complications. Diagnosis is primarily clinical, with imaging studies, such as ultrasonography or computed tomography (CT) scan, serving to confirm the presence and extent of the cyst [2]. Management of congenital ranula cysts in newborns typically involves surgical excision, with intraoral or extraoral approaches depending on the location and size of the cyst. Early diagnosis and treatment are crucial to prevent complications such as infection, feeding difficulties, and airway obstruction [3]. In this article, we present a case of a congenital ranula cyst in a newborn.

## Case presentation

**Figure 1:**
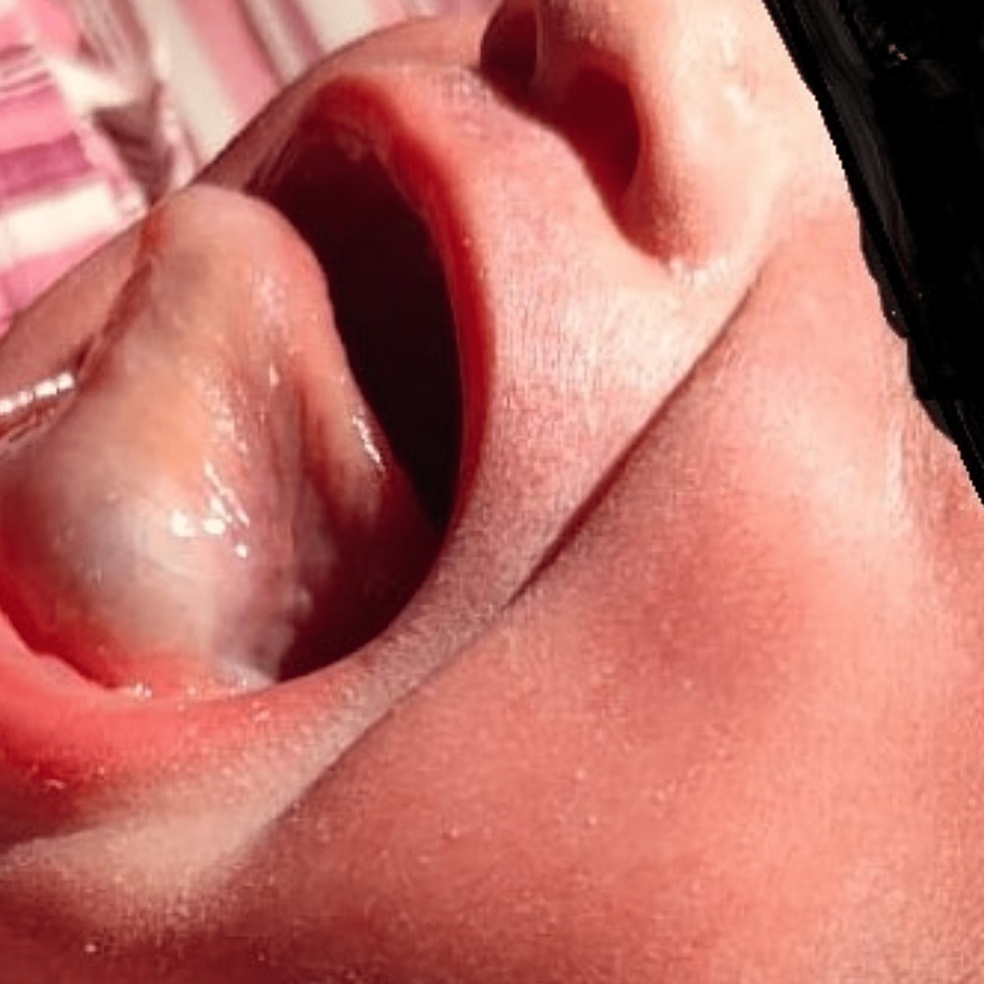
Clinical photograph of the patient with a congenital ranula - swelling originating from the floor of the mouth, almost filling the mouth, and pushing the tongue backward

A male baby weighing 3 kg was born to a G4P2L2A1 (Gravida 4 Para 2 Live 2 Abortion 1) mother via normal vaginal delivery at 38 weeks of gestation. The baby cried immediately after birth but had mild respiratory distress. On examination, a bluish, translucent swelling was observed on the floor of the mouth. The swelling was approximately 2*2 cm in size, round to oval, and had a regular margin with a smooth surface (Figure 1). It was non-pedunculated and non-pulsatile, and it raised the tongue, causing difficulty in feeding and airway obstruction, so the patient was kept nil per oral and intravenous feeding started. Palpation of the swelling revealed it to be soft, non-tender, fluctuant, and cystic. It did not bleed on touch and showed transillumination without any transmitted pulsations.

Based on the clinical and radiological findings, the diagnosis of a congenital ranula cyst was made. The newborn was scheduled for surgical excision under general anesthesia.

**Figure 2:**
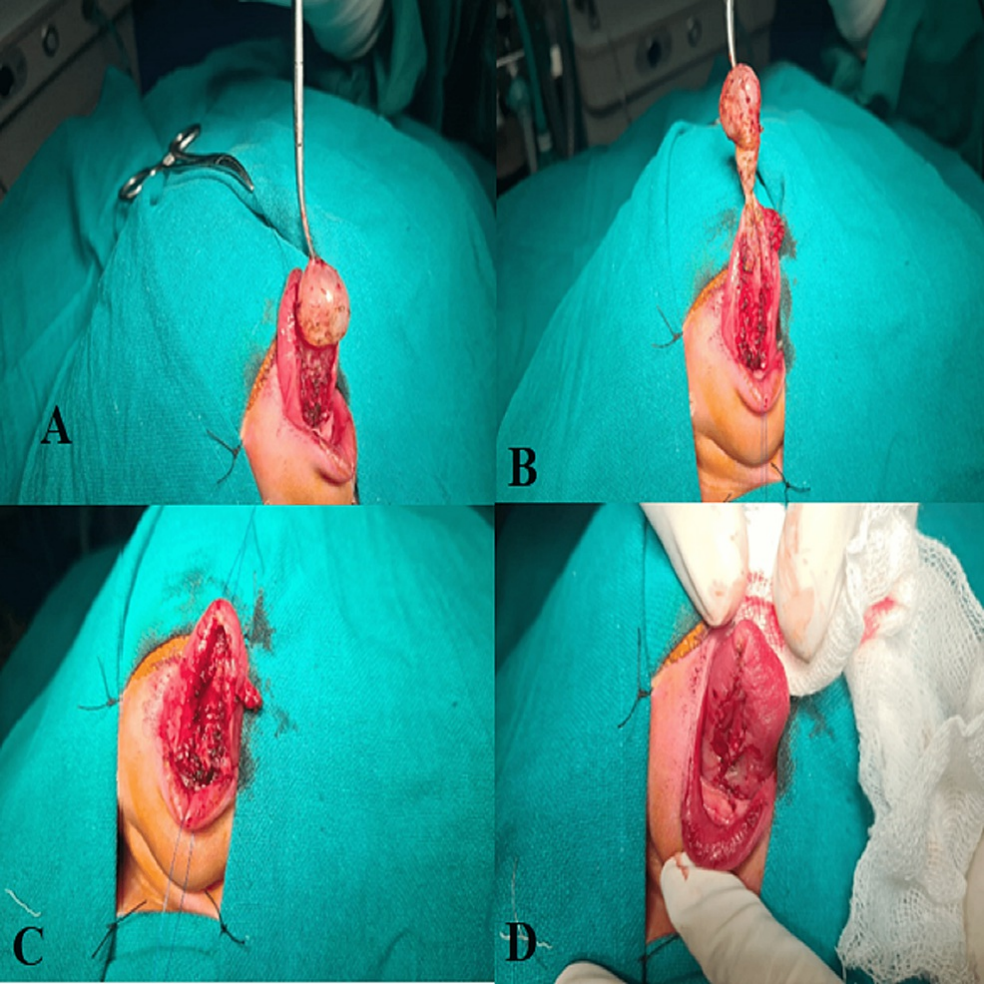
A, B, C, D: intraoperative appearance of the cystic mass

**Figure 3:**
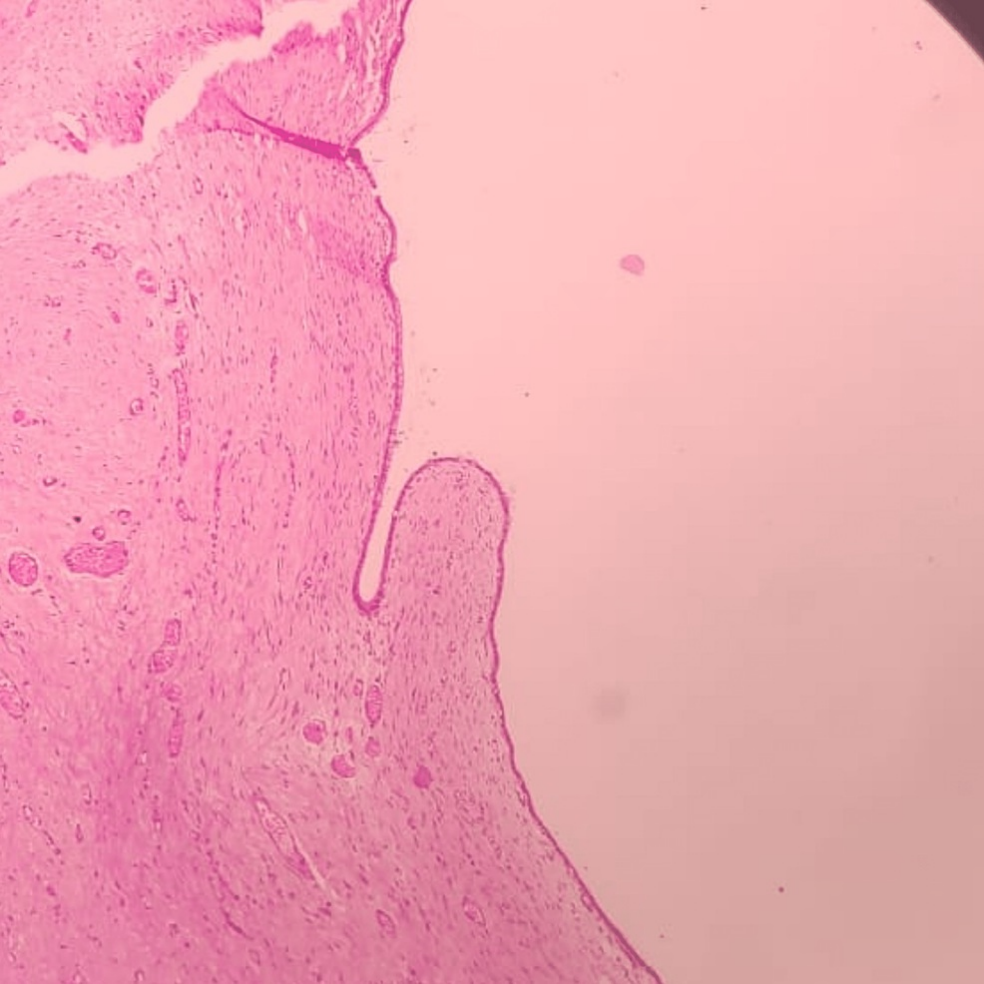
Histopathological picture of the excised ranula showing the cyst wall lined by low columnar to mucinous columnar epithelial cells

During surgery, a transoral approach was used to expose the cyst. The cyst was found to be located in the sublingual space and was connected to the sublingual gland via a stalk. The cyst was carefully dissected from the surrounding tissue and the stalk was ligated (Figure 2). The excised cyst was sent for histopathological examination (Figure 3), which confirmed the diagnosis of a congenital ranula cyst. The postoperative course was uneventful, and the patient was discharged on the fifth day after surgery. A follow-up visit two weeks later revealed complete resolution of the swelling, with no evidence of recurrence.

## Discussion

Congenital ranula cysts are rare, and only a few cases have been reported in the literature. The exact etiology of congenital ranula cysts is not well understood, but it is believed to result from a developmental anomaly in the sublingual gland or duct [4].

The diagnosis of congenital ranula cysts can be challenging, as they can be mistaken for other conditions such as lymphangiomas, dermoid cysts, or thyroglossal duct cysts. Imaging studies, such as CT or magnetic resonance imaging (MRI), can be useful in confirming the diagnosis and identifying any associated anomalies [5].

Surgical excision is the treatment of choice for congenital ranula cysts. The procedure can be performed using a transoral or extraoral approach, depending on the location and size of the cyst. The surgical approach should be carefully planned to minimize the risk of injury to adjacent structures [6].

## Conclusions

Congenital ranula cysts are a rare condition that should be considered in the differential diagnosis of neonatal oral swelling. Diagnosis can be confirmed with imaging studies, and surgical excision is the treatment of choice. A careful surgical approach is required to minimize the risk of complications.
